# Survey, isolation and characterisation of *Bipolaris sorokiniana* (Shoem.) causing spot blotch disease in wheat under the climatic conditions of the Indo–Gangetic plains of India

**DOI:** 10.1016/j.heliyon.2024.e40398

**Published:** 2024-11-14

**Authors:** Sunanda Chakraborty, Sunita Mahapatra, Anubhab Hooi, B. Teja Bhushan, Mansour I. Almansour, Mohammad Javed Ansari, Akbar Hossain

**Affiliations:** a School of Smart Agriculture, Adamas University, Adamas Knowledge City, Barasat - Barrackpore Road, Jagannathpur, Kolkata, West Bengal 700126, India; b Department of Plant Pathology, Survey Selection and Mass Production unit (SSMP), Bidhan Chandra Krishi Viswavidyalaya, Mohanpur, Nadia, West Bengal, 741252, India; c Department of Zoology, College of Science, King Saud University, PO Box -2455, Riyadh, 11451, Saudi Arabia; d Department of Botany, Hindu College Moradabad (Mahatma Jyotiba Phule Rohilkhand University Bareilly), Moradabad, Uttar Pradesh, 244001, India; e Soil Science Division, Bangladesh Wheat and Maize Research Institute, Dinajpur, 5200, Bangladesh

**Keywords:** *Bipolaris sorokiniana*, Spot blotch, Pathogenicity, Morphomolecular studies

## Abstract

Wheat spot blotch has emerged as a disease of significant concern in recent years. The pathogen *Bipolaris sorokiniana* can infect stems, leaves, roots and seeds, increasing its impact. The pathogen is particularly prevalent in the wheat growing zone of West Bengal, which provides congenial conditions for the growth and development of the pathogen. However, its knowledge under West Bengal conditions is inadequate. To address this issue, isolates of *Bipolaris sorokiniana* were collected from different locations in West Bengal. The pathogenic species were identified via comprehensive morphological studies supplemented with internal transcribed spacer (ITS)-rDNA sequence analysis and pathogenicity. The disease severity of the twelve isolates collected varied among the surveyed locations, ranging from 44.03% to 81.48 %. The greatest radial growth was observed in BSC11 (50.07 mm), whereas the lowest growth was recorded in BSC9 (6.47 mm) at 96 h after inoculation. Molecular studies confirmed that the isolates were *Bipolaris sorokiniana*. In pathogenicity assays, BSC11 presented the highest area under the disease progress curve (AUDPC) (380.05) and percent disease index (PDI) among the isolates at 20 dai (38.33 %). The correlation matrix revealed that disease severity was positively correlated with the number of spores (r^2^ = 0.70), growth rate of mycelia (r^2^ = 0.77), and lesion size (r^2^ = 0.74), whereas the length of the spores, incubation period, and latent period were negatively and significantly correlated with disease severity. Establishing the aggressiveness of a pathogen is pivotal in studying host‒ interactions. Our study established BSC11 as a highly virulent pathogen isolate that can be used further for comprehensive analysis of *wheat‒Bipolaris sorokiniana* interactions.

## 1 Introduction

Wheat is one of the most stable crops worldwide, with 35 % of the world's population depending on it for fulfilling their daily caloric intake [1]. According to FAO statistics, approximately 770 million metric tonnes are produced worldwide, representing an area of 221 million hectares [2]. However, a significant increase in wheat production needs to be achieved by 2050 to ensure food security for the ever-increasing world population [3]. Although an array of measures is being taken to maximise the productivity of wheat, minimising yield loss by mitigating biotic and abiotic stresses is still among the foremost concerns [4]. Considering the importance of biomass in increasing productivity and disease resistance, scientists have suggested the role of enhancing biomass by modulating light intensity [5] and increasing nitrogen use efficiency to increase disease resistance [6].

Wheat is susceptible to a variety of pests and diseases that can significantly impact yield and quality. Key pests include aphids, wheat stem sawflies, and armyworms, which can damage plants directly. Diseases such as wheat rust (including leaf, stem, and stripe rust), Fusarium head blight, and powdery mildew can lead to severe losses if not managed effectively. Integrated pest management and disease-resistant varieties are crucial for protecting wheat crops and ensuring sustainable production [7]. Research has been conducted to develop a comprehensive understanding of the host‒pathogen interactions among different pathogens of wheat, such as black points, septoria leaf blotch, and Fusarium head blight [8].

One of the most destructive diseases of wheat, spot blotch disease, causes significant yield loss worldwide [9]. Spot blotch caused by *Bipolaris sorokiniana* leads to significant yield reduction by minimising the active photosynthetic area of the infected plant [10]. The pathogen can infect the leaves, stems, seeds, and roots of plants, increasing its impact [11]. Even a 1 % increase in disease severity significantly reduces the yield of the crop [12]. Although the disease can cause a grain yield reduction of 40–44 % in India, the yield loss can reach 100 % under favourable conditions in susceptible varieties [12].

This disease is more prevalent in warm and humid regions of the world, especially in India [12], the Indo-Gangetic Plains of Bangladesh [13], Pakistan [14] and Nepal [15]. It has also been reported in the wheat-growing regions of Australia [16], South America [17], North Africa [18], West Asia [19], and parts of the United States of America [20]. In India, the northeastern plain zone is particularly susceptible to the disease owing to rising temperatures and occasional drizzling during the winter months [21]. In West Bengal, the districts of Nadia and Murshidabad have been reported to be highly congenial to spot blotch development and have recorded high disease severity in recent years. A disease severity of 59.26 % was recorded in Murshidabad in 2020–21 [22].

The pathogen *Bipolaris sorokiniana* is a hemibiotroph that initially infects living tissues but switches to a necrotrophic mode once inside its host [9]. It is highly diverse, with a range of colony colours, textures, growth rates and virulence among its isolates [23]. However, species identification exclusively on the basis of morphological studies might lead to misidentification of the pathogen owing to phenotypic plasticity and overlapping morphology [24]. For the molecular diversity analysis and characterisation of *Bipolaris sorokiniana* isolates, PCR-based universal rice primers [23] and primers based on variations in the internal transcribed spacer (ITS) region [25] have been used previously.

According to morpho-virulence studies, management strategies also vary in terms of the time and quantity of application. Additionally, the variation in pathogenicity dictates the aggressiveness of management strategies [26]. Thus, establishing an association between the morphological attributes and virulence of the pathogen, if any, is imperative.

The present study was thus conducted with the purpose of determining the extent of variability and pathogenicity among the isolates collected from spot blotch hotspots in West Bengal. Therefore, this study focused on the following objectives: (1) to determine the disease severity of *Bipolaris sorokiniana* isolates in spot blotch hotspots in West Bengal; (2) to study the morphomolecular variability among the isolates; (3) to assess the pathogenicity of the collected isolates; and (4) to study the associations between the morphological attributes and virulence of the isolates.

## 2 Materials and methods

### 2.1 Roving survey in different locations of West Bengal

A survey of rovings was conducted at twelve locations across two districts of West Bengal, India, viz., Nadia and Murshidabad. The districts were chosen owing to their reportedly high disease severity over the years to spot blotch disease. Disease severity was recorded according to the modified Saari–Prescott (00–99) scale [27,28]. Here, the first digit (D1) indicates the diseased leaf area in the flag leaf (F), and the second digit (D2) refers to the diseased leaf area in the penultimate leaf (F-1). Disease severity was recorded for the plants in the middle rows to avoid buffer effects. Disease severity was calculated via the following formula (Eq. (1)):

The severity % = (D1/9) × (D2/9) × 100 … … … … … … … …. (Eq. (1))

### 2.2 Collection, isolation, and maintenance of fungal samples

Spot blot-infected leaf samples were collected from twelve different locations in West Bengal. The infected leaf samples were placed in labelled plastic bags and taken to the laboratory of the Department of Plant Pathology, Bidhan Chandra Krishi Viswavidyalaya, Nadia, West Bengal, for isolation of the pathogen. The infected leaves were cut into 5 mm squares with approximately 50 % healthy tissue. The processed samples were surface sterilised by dipping in 1 % sodium hypochlorite solution (NaOCl) (v/v) for 60 s, followed by rinsing three times with sterile distilled water. The samples were then transferred to sterilised tissue paper and plated onto sterile potato dextrose agar media. Three bits were placed on one plate, with three replications per sample. The media was supplemented with streptomycin to avoid bacterial contamination. The plates were wrapped with parafilm and incubated at 27 ± 1 °C. Mycelial growth was transferred to a fresh plate containing potato dextrose agar (PDA) media via the hyphal tip technique, and monoconidial cultures were obtained via the single spore technique [29]. Morphomolecular characterisation of the pathogen was conducted following successful establishment of the pathogen in pure culture.

### 2.3 Morphological characterisation of the pathogen

Morphological characterisation of the pathogen colony and the fungal spores was performed. The colony characteristics, i.e., color, texture, and growth type, of the colonies were assessed by placing a 1 cm disc of purified fungal culture in the center of the PDA plates, sealing it with parafilm and incubating it at 27 ± 1 °C. The colony growth rate was measured at 24, 48, 72, 96 and 120 h after inoculation, as described by Chauhan et al. [30]. The mean growth rate was calculated as an average of the observed colon growth (mm/day). Colony pigmentation and texture were measured in 7-day-old cultures.

The microscopic features of the pathogen were assessed in 10-day-old cultures. The cultures were washed with sterile distilled water and observed via a Carl Zeiss microscope (10X and 40× magnifications). The conidial characteristics were studied using 25 mature conidia from each of the isolates. The description given by Mew and Gonzales [31] was followed for pathogen identification.

### 2.4 Molecular characterisation

For molecular characterisation of the pathogen, the purified fungal culture was transferred to potato dextrose broth at 27 °C in a shaker incubator. After seven days, the fungal mat was collected on sterilised Whatman filter paper under aseptic conditions and stored at −20 °C until further processing. Liquid nitrogen was poured onto the fungal mats, and the samples were crushed using a sterile mortar and pestle. Following homogeneous crushing of the sample, the fungal DNA was extracted via a kit (GSure DNA extraction kit). The quality of the extracted genomic DNA was assessed on a 1 % agarose gel via gel electrophoresis. The extracted DNA was stored at −20 °C for further studies.

### 2.5 PCR amplification

For amplification of the genomic DNA, the universal eukaryotic primers ITS1/ITS4 were used. Amplification was conducted via primers in a thermal cycler (Applied Biosystems 9700). The reaction mixture was prepared with 12.5 μl of master mix (GCC PCR master mix), 7.5 μl of sterile double distilled water, 3 μl of sample DNA, 1 μl of forward primer and 1 μl of reverse primer in 0.2 ml PCR tubes. PCR was performed under the following conditions: an initial denaturation step at 94 °C for 2 min, followed by 35 cycles of denaturation at 94 °C for 45 s, annealing at 59 °C for 45 s, extension at 72 °C for 90 s, and a ﬁnal extension step at 72 °C for 10 min. The amplified PCR products were observed on a 1 % agarose gel in 1X TBE Buffer and visualised under a gel documentation unit (Bio-Rad, USA) with ethidium bromide staining. The size of the PCR product was determined by comparison with a 50 bp ladder. The extracted DNA was subsequently quantified spectrophotometrically in a NanoDrop spectrophotometer with a 1 μl sample. The amplified PCR product was sequenced via the Sanger dideoxy sequencing method (GCC Biotech Pvt. Ltd., Kolkata). The obtained sequences were deposited in the NCBI nucleotide database provided by the National Center for Biotechnology Information, USA, through the GenBank submission tool, and their accession numbers were obtained.

### 2.6 ITS region phylogenetic analyses

The nucleotide sequences were retrieved from NCBI GenBank (https://www.ncbi.nlm.nih.gov; accessed on April 17, 2024) and compared for similarity or diversity. The sequences were searched against the NCBI database via the Basic Local Alignment Search Tool for Nucleotide Sequences (BLASTn). Multiple sequence alignment of the ITS sequences of *Bipolaris sorokiniana* isolates was conducted via the ClustalW algorithm integrated in MEGA6 software. The pairwise sequence alignment was conducted with BioEdit [32]. Phylogenetic trees were reconstructed via the neighbor-joining (NJ) algorithm [33] and maximum likelihood (ML) methods in MEGA version 6 [34].

### 2.7 Pathogenicity assessment

The collected isolates of *Bipolaris sorokiniana* were subjected to pathogenicity assessment under controlled conditions during the rabi season of 2021–22 via the susceptible variety K 1317 obtained from the IIWBR, Karnal. To prepare the inoculum, the pathogen was mass cultured on sterilised wheat seeds by inoculating them with fungal discs under aseptic conditions. The inoculated flasks were incubated at 27 °C for 20–30 days, with regular mixing at seven-day intervals to ensure homogeneous growth of the fungus. Upon complete growth of the inoculated seeds, sterile distilled water was added to the flasks, which were subsequently sieved through three layers of muslin cloth. The spore count was adjusted to 10^4^ using a hemocytometer. Tween 20 was added to the suspension to ensure adherence and even distribution of the spores on the leaves [23].

For pathogenicity assessment, seeds were sown in pots (12 cm diameter) containing nutrient-rich soil inside the net house of the Department of Plant Pathology, Bidhan Chandra Krishi Vishwavidyalaya, West Bengal. N:P:K was added at a ratio of 120:60:60 to the soil as a basal dose during soil preparation. The seeds were sown at a depth of 6 cm, followed by light irrigation to encourage seed germination. Three replications of each treatment were performed to reduce error. The leaf was inoculated with the pathogen suspension at the two-leaf stage GS13 of the Zadoks growth stage [35] via a hand sprayer until run-off [36]. The inoculated plants were transferred to a humid chamber (100 % RH) to provide favourable conditions for pathogen growth. The plants were then transferred to a net and observed regularly for the development of symptoms.

Virulence assessment of the isolates was conducted via a 1–5 severity scale where 1 = no visible symptoms/chlorotic flecks; 2 = up to 10 % leaf area covered with small, restricted lesions; 3 = 11–25 % leaf area covered with small, restricted lesions; 4 = 26–50 % leaf area covered with large coalescing lesions; and 5 = >50 % leaf area covered with large coalescing lesions. Assessment was performed 3–20 days after inoculation. Disease severity was converted into the percent disease index (PDI) via the formula given by McKinney [37]. The area under the disease progression curve (AUC) was calculated via the formula developed by Campbell and Madden [38]. The partial disease resistance (PDR) components, *viz.,* the incubation period (days), latent period (days), and lesion size, were recorded as suggested by Bashyal et al. [39].

### 2.8 Statistical analyses

All the statistical analyses were conducted via IBM SPSS (version 20.0 SPSS Inc., Chicago IL, USA). The visual representation of the data was performed via the R software package (version 4.2.2 (2022–10–31 ucrt)).

## 3 Results

### 3.1 Roving survey in different locations of West Bengal

A roving survey was conducted at 12 wheat spot blot locations across 2 districts of West Bengal. The districts of Nadia and Murshidabad were chosen owing to their status as hotspots for spot blotch of wheat [22]. Among the locations surveyed, Domkol-Taraf had the greatest disease severity (81.48 %), whereas the lowest DLA was observed in Ghaat More, the Krishnanagar-Karimpur route (44.03 %) (Table 1).

**Table 1:** Disease severity (DLA) of spot blotch in different locations surveyed.

Isolate	Location	Latitude	Longitude	DLA%
BSC1	Kalyani	N 22°58′30.3024″	E 88° 26′ 4.2324″	69.14^ab^ ± 3.28
BSC2	Karimpur	N 23°26′50.2692	E 88°31′51.762	51.85^cd^ ± 12.83
BSC3	Mahatpur	N 23°28′21.4608	E 88°32′28.2444	68.72^ab^ ± 6.42
BSC4	Bangaljhi	N 23°30′15.6528	E 88°33′13.95	71.60^ab^ ± 10.69
BSC5	Arangsarisha	N 23°33′09.5364	E 88°32′12.6348	61.73^bc^ ± 4.28
BSC6	Lakshmigacha	N 23°34′53.508	E 88°31′23.7932	60.91^bc^ ± 9.98
BSC7	Taranipur	N 23°39′6472	E 88°31′41.4804	54.32^cd^ ± 4.28
BSC8	Puthimari	N 23°40′13.0332	E 88°31′38.3376	61.73^bc^ ± 4.28
BSC9	Ghaat more, Krishnanagar - Karimpur route	N 23°19.2323	E 88°31′57.3132	44.03^d^ ± 7.44
BSC10	Tehatta	N 23°330.7212	E 88°3218.6612	59.26^bc^ ± 7.41
BSC11	Domkol-Taraf	N 24°5238.3232	E 88° 3351.8148	81.48^a^ ± 9.27
BSC12	Jalangi	N 24° 10′ 3.198″	E 88° 16′ 14.9484″	77.78^a^ ± 3.28

^1^Numbers with the same superscripted letters are significantly parallel to each other according to the Duncan multiple range test.

### 3.2 Morphological characterisation

**Fig. 1:**
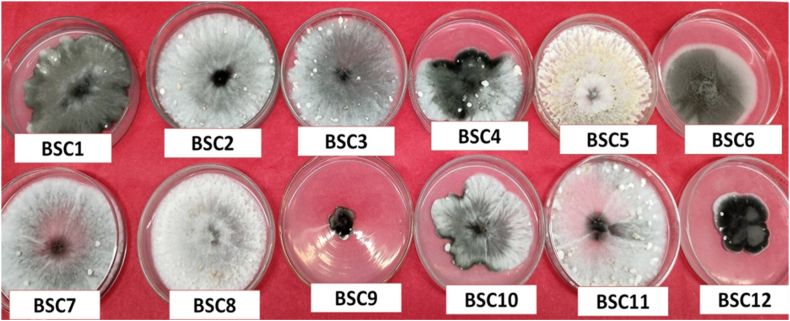
Morphological study of twelve isolates of *Bipolaris sorokiniana* collected from different locations in West Bengal.

A total of 12 *Bipolaris sorokiniana* isolates were collected and subjected to morphological characterisation. The isolates exhibited significant variability with respect to mycelial growth, colony margins, colony color, and conidial shape on PDA media (Fig. 1 and Table 2). Among the isolates, the colonies were white, yellowish white, whitish grey, grayish white, dark grey and black. The colony margins were either regular or irregular among the isolates, while the texture ranged from cottony to velvety.

**Table 2:** Morphological characterisation of different isolates of *Bipolaris sorokiniana* on PDA media on the 10th day.

Isolates	Colony color	Mycelial growth	Margin of colony	Conidial shape	Sporulation
BSC1	Dark grey with black center	Thick growth without zonation	Irregular	Oblong	+++
BSC2	Greyish white with black center	Thick growth without zonation	Regular	Elliptical	+++
BSC3	Whitish grey with grey center	Thick velvety growth without zonation	Regular	Slightly curved	+++
BSC4	Greyish white with black center	Black zonation	Irregular	Slightly curved	+++
BSC5	Yellowish white	Cottony growth	Irregular	Oblong	++++
BSC6	Dark grey with light grey margin	Velvety growth	Regular	Oblong	+++
BSC7	Greyish white with black center	Cottony growth	Regular	Oblong	+++
BSC8	White with grey zonations	Cottony growth	Regular	Slightly curved	++++
BSC9	Black colony with light grey margin	Velvety growth	Irregular	Oblong	+
BSC10	Greyish white with black center	Velvety growth	Irregular	Oblong	++
BSC11	Greyish white with black center	Cottony growth	Regular	Elliptical	++++
BSC12	Black colony with light grey margin	Velvety growth	Irregular	Oblong	+

Mycelial growth was observed at 24, 48, 72 and 96 h after inoculation (hai) on PDA plates, and the average growth rate was calculated (mm/day). The growth rates were analysed statistically and observed to differ significantly among the isolates. Furthermore, the mycelial growth rate of different isolates fluctuated at different intervals after inoculation, with some isolates growing faster initially and slowing down eventually with an increase in hours after inoculation (Table 3).

**Table 3:** Differences in the radial growth (mm) of different isolates at 24, 48, 72 and 96 h after inoculation (hai) and the growth rate (mm/day) of *Bipolaris sorokiniana*.

Isolate	24hai	48hai	72hai	96hai	Growth rate
BSC1	6.20^e^	13.47^e^	20.67^e^	38.03^c^	7.23 ± 0.14^cd^
BSC2	7.30^c^	15.23^cd^	23.50^bc^	39.77^c^	8.10 ± 0.28^a^
BSC3	7.13^cd^	14.80^d^	22.20^d^	38.80^c^	7.53 ± 0.18^bc^
BSC4	2.63^f^	4.63^f^	8.37^f^	16.60^d^	2.87 ± 0.10^e^
BSC5	6.90^cd^	13.63^e^	21.10^e^	38.40^c^	7.10 ± 0.31^d^
BSC6	6.63^de^	13.27^e^	20.33^e^	38.10^c^	6.85 ± 0.23^d^
BSC7	8.67^b^	16.37^b^	24.20^ab^	45.70^b^	7.77 ± 0.06^ab^
BSC8	9.60^a^	17.60^a^	24.87^a^	46.60^b^	7.63 ± 0.39^b^
BSC9	1.23^h^	3.13^g^	3.43^h^	6.47^f^	1.10 ± 0.13^g^
BSC10	2.80^f^	4.70^f^	8.63^f^	17.67^d^	2.92 ± 0.21^e^
BSC11	8.33^b^	15.97^bc^	22.70^cd^	50.07^a^	7.18 ± 0.14^cd^
BSC12	2.00^g^	3.67^g^	6.73^g^	12.00^e^	2.37 ± 0.15^f^
S.E.m(±)	0.10	0.18	0.22	0.57	0.12
C.D. (5 %)	0.30	0.53	0.63	1.68	0.36

The numbers with the same superscript letters are significantly more parallel to each other according to Duncan's multiple range test.

**Fig. 2:**
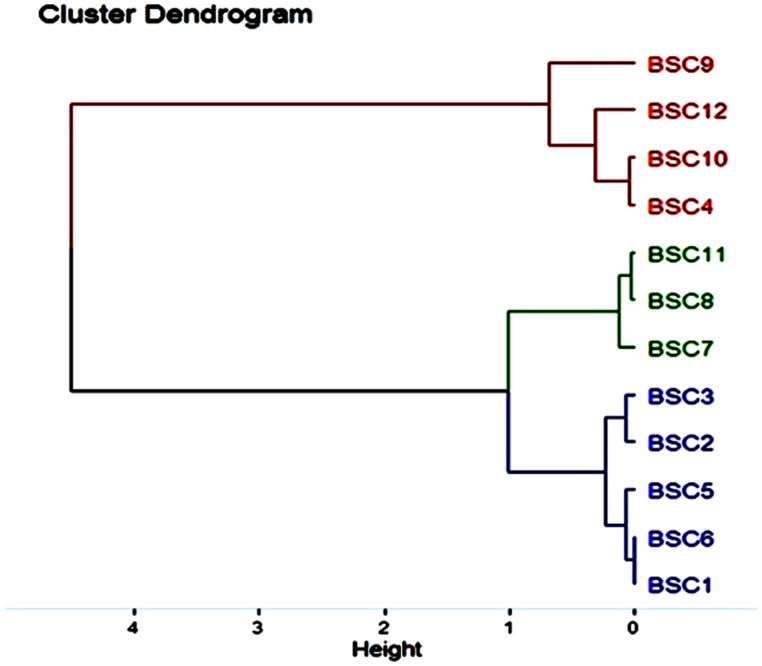
Morphological dendrogram of different isolates of *Bipolaris sorokiniana* on the basis of growth rate via hierarchical clustering.

Hierarchical clustering was performed on the basis of the growth rate. The isolates were clustered into two groups with 4 % Euclidean distance: Group 1 consisted of isolates with growth rates greater than 6 mm/day, whereas Group 2 consisted of isolates with considerably lower growth rates between 1 mm/day and 3 mm/day (Fig. 2).

The variability in spore morphology was assessed in a 10-day-old culture via a Carl Zeiss microscope at 10X and 40× magnifications (Table 4). The average conidial length ranged from 37.71 ± 0.74 μm (BSC11) to 73.53 ± 4.50 μm (BSC1), whereas the average conidial width ranged from 16.59 ± 2.38 μm (BSC8) to 21.21 ± 1.77 μm (BSC1). The dimensions were observed to vary significantly among the isolates assessed. Additionally, the isolates exhibited variability with respect to the number of septations, with isolate BSC8 (9.67) having the greatest number of septa and isolate BSC11 (3.33) having the least number of septations (Table 4).

**Table 4:** Variations in size and sporulation of different isolates of *Bipolaris sorokiniana*.

Isolate	Length (μm)	Width (μm)	No. of Spores	Septation
BSC1	73.53 ± 4.50^a^	21.21 ± 1.77^a^	225.25 ± 20.5^cd^	9.33^a^
BSC2	37.75 ± 1.08^f^	19.65 ± 1.20^abc^	261.19 ± 21.24^bc^	4.00^e^
BSC3	62.17 ± 5.38^bcd^	21.09 ± 0.61^a^	235.29 ± 45.32^c^	5.67^d^
BSC4	54.99 ± 2.28^de^	19.56 ± 2.00^abc^	266.05 ± 21.94^abc^	7.33^c^
BSC5	54.19 ± 8.34^de^	17.59 ± 1.61 ^bc^	335.86 ± 26.4^a^	5.67^d^
BSC6	57.12 ± 2.19^cde^	18.26 ± 0.9 ^abc^	268.60 ± 23.15^ab^	8.67^ab^
BSC7	63.95 ± 2.58^bc^	18.92 ± 2.76^abc^	280.65 ± 12.28^abc^	7.33^c^
BSC8	54.13 ± 6.78^de^	16.59 ± 2.38^c^	312.49 ± 18.67^ab^	9.67^a^
BSC9	73.45 ± 3.69^a^	20.13 ± 0.53^ab^	94.07 ± 2.62^e^	7.67^bc^
BSC10	69.34 ± 3.19^ab^	19.58 ± 0.99^abc^	159.47 ± 23.96^de^	5.33^d^
BSC11	37.71 ± 0.74^f^	19.80 ± 1.12^abc^	331.16 ± 33.72^ab^	3.33^e^
BSC12	49.22 ± 4.29^e^	19.30 ± 2.54^abc^	92.91 ± 3.43^e^	5.33^d^
S.E.m(±)	2.502	0.534	13.813	0.36
C.D. (5 %)	7.348	1.664	40.557	1.06

The numbers with the same superscript letters are significantly more parallel to each other according to Duncan's multiple range test.

### 3.3 Molecular characterisation

**Fig. 3:**
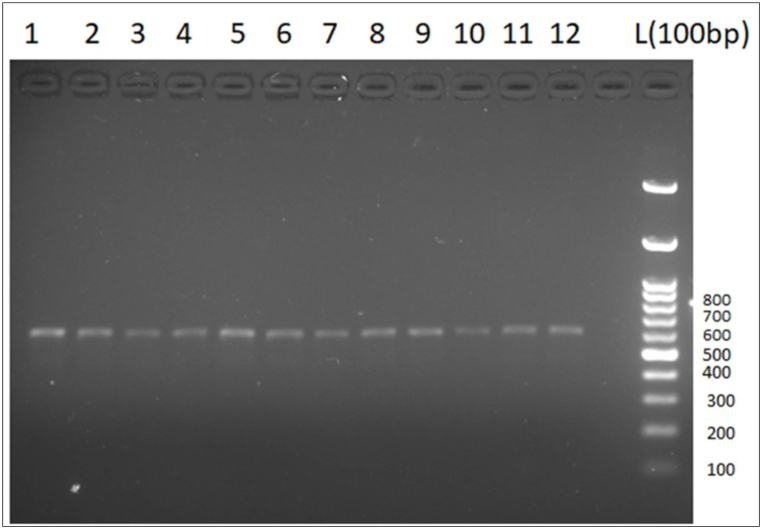
PCR amplification of the ITS region of 12 *Bipolaris sorokiniana isolates*.

The isolates collected during the study were subjected to morphomicroscopic characterisation and further validated via molecular identification. Amplification of the ITS region was performed via the ITS-specific primer pairs ITS1 and ITS4, which produced an amplified product of 600 fragments (Fig. 3).

BLAST analysis of the ITS rDNA sequences revealed correspondence with the morphomicroscopic characterisation of the isolates. The isolates shared 98–99 % genetic similarity with *Bipolaris sorokiniana*. The ITS rDNA sequences were deposited in the NCBI GenBank database with distinct accession numbers (Table 5).

**Table 5:** Identification of *Bipolaris sorokiniana* isolates on the basis of ITS sequences through a BLASTn search of the GenBank database.

Isolates	Length of ITS sequenced	Gene bank accession number	Most closely related organisms				
			ITS identification	NCBI accession description	%Gene identity	% query coverage	E value
BSC1	633	OR188298	*Bipolaris sorokiniana* isolate PJC5	OM419194	98.48	93	0.0
BSC2	628	OR188339	*Bipolaris sorokiniana* isolate A82	OQ225212	99.66	93	0.0
BSC3	600	OR188351	*Bipolaris sorokiniana* isolate CBS120.24	KJ909776	98.26	95	0.0
BSC4	629	OR188406	*Bipolaris sorokiniana* isolate M21	MH592543	98.98	93	0.0
BSC5	629	OR188576	*Bipolaris sorokiniana* isolate A47	OQ225217	99.00	95	0.0
BSC6	613	OR188683	*Bipolaris sorokiniana* isolate WLB-18-259	OM023025	98.64	93	0.0
BSC7	627	OR188703	*Bipolaris sorokiniana* isolate C109-13	MN313291	99.33	95	0.0
BSC8	631	OR188716	*Bipolaris sorokiniana* culture CBS:127724 strain CBS 127724	MH864698	98.68	95	0.0
BSC9	629	OR188776	*Bipolaris sorokiniana* isolate C404-11	MN313300	99.01	96	0.0
BSC10	633	OR188815	*Bipolaris sorokiniana* isolate 83	MN534829	98.37	96	0.0
BSC11	629	OR188843	*Bipolaris sorokiniana* strain LK93 chromosome 12	CP102792	98.88	99	0.0
BSC12	628	OR188842	*Bipolaris sorokiniana* strain HB716	MT664148	98.69	97	0.0

**Fig. 4:**
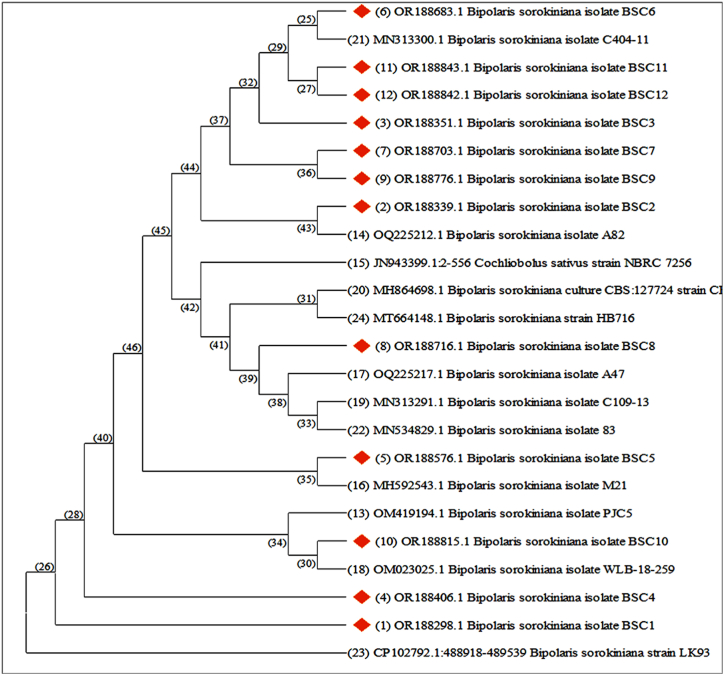
Neighbor-joining tree of *Bipolaris sorokiniana* isolates based on ITS region sequences.

ITS sequences of *Bipolaris sorokiniana* isolates BSC1-- BSC12 were aligned with the consensus region via the CLUSTAL W program. A total of 1000 bootstrap replicates were performed, and the bootstrap replication percentages are given in the internal nodes of the phylogenetic tree. The topologies of the neighbor‒joining tree were constructed with the CLC sequence viewer. The phylogenetic tree used all twelve isolates collected as well as twelve reference sequences retrieved from the NCBI database. Phylogenetic analysis grouped *Bipolaris sorokiniana* isolates into multiple clusters (Fig. 4).

### 3.4 Pathogenicity assessment

**Fig. 5:**
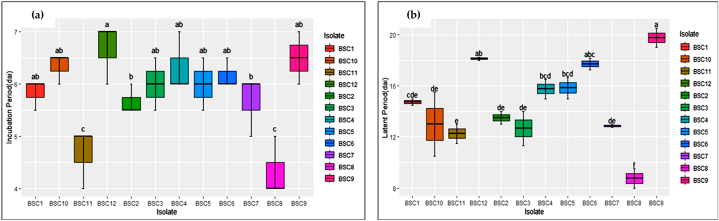
(a) Incubation period; (b) Latent period in days after inoculation (dai) of twelve isolates of *Bipolaris sorokiniana* after artificial inoculation of wheat under controlled conditions.

Pathogenicity tests of the twelve isolates of *Bipolaris sorokiniana* confirmed that the isolates were causal organisms of spot blotch in wheat and exhibited various degrees of virulence to the wheat variety K 1317. The experiment was conducted in the net house of the Department of Plant Pathology, Bidhan Chandra Krishi Viswavidyalaya, West Bengal. The wheat crop was artificially inoculated with the pathogen suspension and incubated under favourable conditions. The inoculated plants started developing symptoms at 8 days after inoculation. Characteristic yellow spots were observed on inoculated plants that later became necrotic, with yellow discolouration. The percent disease index was recorded on a 1–5 scale (Table 6). The pathogenicity of the isolates was further confirmed by validating Koch's postulates. Partial disease resistance components, *viz.*, the incubation period (Fig. 5(a)), latent period and lesion size (Fig. 5(b)), were also recorded.

**Table 6:** Assessment of the pathogenicity of twelve isolates of *Bipolaris sorokiniana* on wheat.

Isolate	Percent Disease Index				AUDPC	Lesion size (mm)
	8 dai	12 dai	16 dai	20 dai		
BSC1	6.20^e^	13.47^e^	6.57^b^	38.03^c^	281.71	7.23 ± 0.14^cd^
BSC2	7.30^c^	15.23^cd^	7.50^a^	39.77^c^	302.09	8.10 ± 0.28^a^
BSC3	7.13^cd^	14.80^d^	7.27^ab^	38.80^c^	261.68	7.53 ± 0.18^bc^
BSC4	2.63^f^	4.63^f^	5.23^c^	16.60^d^	250.29	2.87 ± 0.10^e^
BSC5	6.90^cd^	13.63^e^	7.27^ab^	38.40^c^	269.74	7.10 ± 0.31^d^
BSC6	6.63^de^	13.27^e^	6.43^b^	38.10^c^	276.08	6.85 ± 0.23^d^
BSC7	8.67^b^	16.37^b^	7.70^a^	45.70^b^	319.67	7.77 ± 0.06^ab^
BSC8	9.60^a^	17.60^a^	8.17^a^	46.60^b^	345.10	7.63 ± 0.39^b^
BSC9	1.23^h^	3.13^g^	1.70^e^	6.47^f^	222.74	1.10 ± 0.13^g^
BSC10	2.80^f^	4.70^f^	5.20^c^	17.67^d^	262.62	2.92 ± 0.21^e^
BSC11	8.33^b^	15.97^bc^	7.93^a^	50.07^a^	380.05	7.18 ± 0.14^cd^
BSC12	2.00^g^	3.67^g^	3.27^d^	12.00^e^	254.09	2.37 ± 0.15^f^
S.E.m(±)	0.10	0.18	0.29	0.57		0.12
C.D. (5 %)	0.30	0.53	0.86	1.68		0.36

The numbers with the same superscript letters are significantly more parallel to each other according to Duncan's multiple range test.

**Fig. 6:**
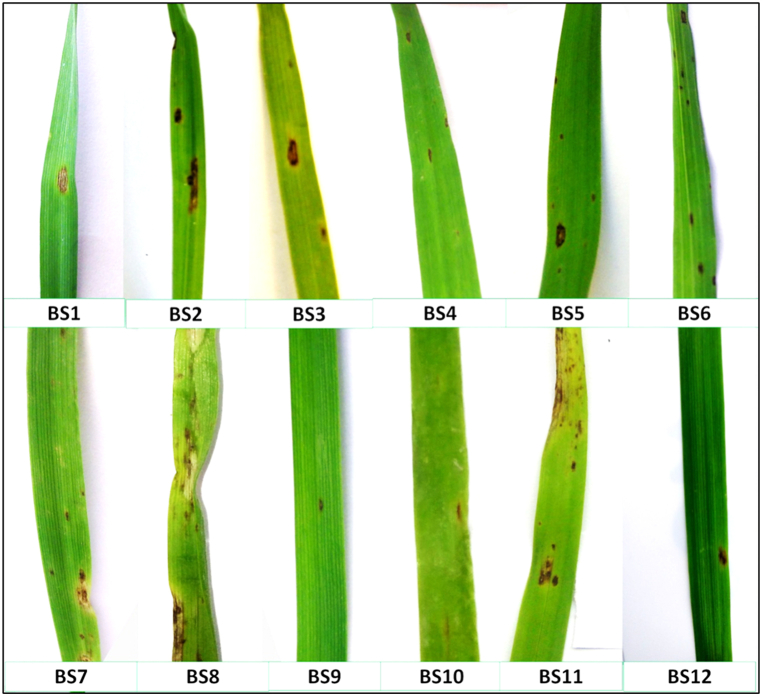
Disease symptoms exhibited by isolates of *Bipolaris sorokiniana* on wheat leaves after artificial inoculation under controlled conditions.

The disease severity of the isolates ranged from 3.33 % (BSC9) to 18.33 % (BSC11) at 8 days after inoculation (Fig. 6). The percent disease index increased gradually with increasing days after inoculation, with the highest value recorded at 20 days after inoculation (dai). At 20 dai, the BSC11 (38.33 %) and BSC9 (18.33 %) isolates presented the highest and lowest percent disease indices, respectively. The area under the disease progress curve (AUDPC) was calculated for each isolate on the basis of the percent disease index. The maximum AUDPC was observed in BSC11 (380.05), whereas the minimum AUDPC value was recorded in BSC9 (222.74). In terms of AUDPC, the isolates were arranged in the following order: BSC11 > BSC8 > BSC7 > BSC2 > BSC1 > BSC6 > BSC5 > BSC10 > BSC3 > BSC12 > BSC4 > BSC9. Additionally, the lesion size varied significantly in plants inoculated with different isolates of the pathogen. The maximum lesion size was recorded for BSC8 (8.17 mm), whereas the minimum lesion size was observed for BSC9 (1.70 mm).

When the incubation periods (IP) of the different isolates were recorded, the maximum and minimum values were observed for BSC12 (6.67 days after inoculation) and BSC8 (4.33 days after inoculation), respectively. Additionally, the duration from symptom onset to spore development was observed to be an average of 4 days among the isolates. The maximum latent period was 19.75 days after inoculation (BSC9), whereas the minimum latent period was 8.75 days after inoculation (BSC8) (Fig. 5).

### 3.5 Correlation study

**Fig. 7:**
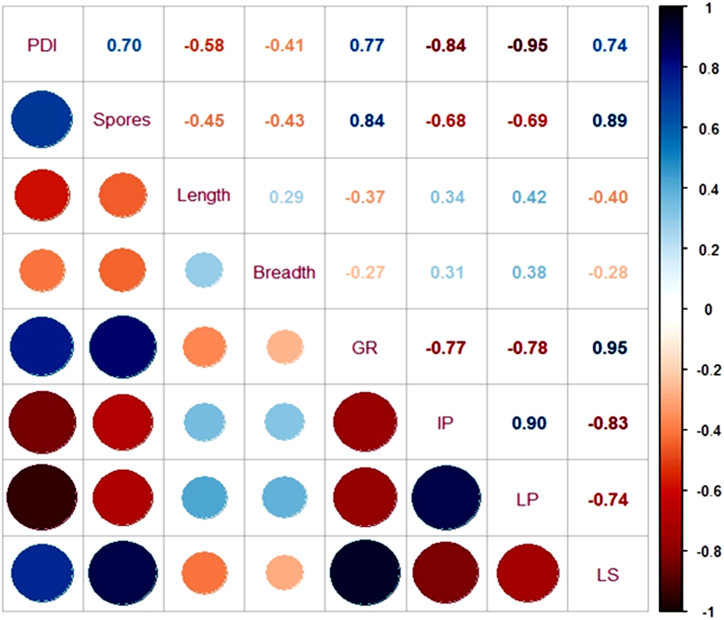
Correlation between the percent disease index (PDI%) and pathogen attributes, viz., spores produced, conidial length, conidial breadth, radial growth (GR), incubation period (IP), latent period (LP) and lesion size (LS).

A correlation matrix was drawn between disease severity and 8 components of *Bipolaris sorokiniana* (*Bs*), such as the number of spores, spore length, spore breadth, growth rate, incubation period, latency period and lesion size (Fig. 7). The correlation matrix revealed that disease severity was positively correlated with the number of spores (r^2^ = 0.70), growth rate of mycelia (r^2^ = 0.77), and lesion size (r^2^ = 0.74), whereas the length of the spores, incubation period, and latent period were negatively and significantly correlated with disease severity.

## 4 Discussion

Wheat has been a predominant staple worldwide for thousands of years. However, crops are susceptible to a plethora of biotic stresses, which negatively affect yield globally [40].

One of the most destructive pathogens that attacks the stems, leaves, and seeds of wheat is *Bipolaris sorokiniana* [41]. This pathogen has been associated with spot blotch of wheat in numerous studies, with losses ranging from 16 to 43 %, especially in warm and humid regions of the world [42]. In India, the pathogen is of major importance in eastern wheat growing zones owing to warmer temperatures and erratic drizzling during the winter months [21,43].

The management of a disease focuses largely on a combination of strategies [44], including the study of biochemical parameters [45], the use of biopesticides [46,47], and the use of botanicals [48]. However, the most pivotal step towards disease management is a comprehensive understanding of the associated pathogen.

In the course of the study, we identified twelve isolates of *Bipolaris sorokiniana* from twelve different locations spanning two districts of West Bengal. The disease severity varied across the locations surveyed. The prevalent meteorological factors, different sowing times, and use of different diseases resistant to tolerant varieties in the area might have influenced the variation in the degree of disease development. This phenomenon was previously identified by Tamang et al. [49], who elucidated the role of abiotic factors in spot blot disease development in wheat. However, the virulence assessment confirmed the influence of varying the aggressiveness of the isolates on the variation in disease severity in the surveyed locations. Our results are in keeping with the findings of Hooi et al. [22], who identified host‒ interactions as major determinants of disease development in *wheat‒Bipolaris sorokiniana* interactions.

Morphomicroscopic characterisation plays a pivotal role in the preliminary identification of a pathogen [50]. In the present study, the isolates were assessed qualitatively on the basis of their colony color, nature of mycelial growth and texture, and colony margin, whereas quantitative assessment was conducted on the basis of mycelial growth and growth rate of the pathogen. The isolates presented an array of colony colours, *viz.,* white, yellowish white, whitish grey, greyish white, dark grey and black. These isolates were grouped into two clusters on the basis of their growth attributes in artificial media. The colony characteristics were similar to those reported by Verma et al. [23]. They also reported similar textural and chromic variations in colony attributes among isolates, which were collected during the course of their study. The variation in morphomicroscopic parameters was also reflected in the study by Devi et al. [51], who reported a variation in growth attributes as well as biochemical parameters among different isolates of *Bipolaris sorokiniana.*

Additionally, the growth of fungal mycelia contributes to disease development. The isolates with relatively high growth rates presented relatively high percent disease indices (PDIs) under controlled conditions. Since *Bipolaris sorokiniana* infects host cells with the help of invading hyphae, the rapid growth rate facilitated faster infection of the host cells, which led to increased manifestation of the disease.

The results of virulence and pathogenicity studies revealed that all the isolates were pathogenic to wheat. However, there was significant variation in the disease severity recorded on the host plant following inoculation with different isolates (Table 6). The isolates were clustered on the basis of the percent disease index, incubation period and latent period. Two major clusters were formed: BSC8 and BSC11 were grouped into one cluster, whereas the remaining isolates were grouped into another cluster.

Substantial differences were observed in the duration required for each isolate to produce symptoms on the inoculated host. Additionally, the latent period also varied among different isolates of *Bipolaris sorokiniana.* The incubation period and latent period are crucial factors in determining the aggressiveness of a pathogen towards its host. Chauhan et al. [52] identified *Bipolaris sorokiniana* isolate BS 4 as the most virulent isolate among the isolates collected on the basis of the incubation period (IP). Our results are consistent with the findings of Mahapatra et al. [27], who reported variation in the IP and LP values across isolates of *Bipolaris sorokiniana*. Partial disease resistance components, such as leaf tip necrosis and lesion mimics, play a significant role in determining the host preference of *Bipolaris sorokiniana* [53].

The isolation of pathogens from infected tissues of the host is not a definitive method, as it might lead to the isolation of endophytes instead of the pathogen [54]. Therefore, it is imperative to conduct a pathogenicity assessment to establish the authenticity of the pathogen as the causal organism of the disease. In the present study, pathogenicity assessment was conducted under controlled conditions via artificial inoculation of the isolates into the host. All the isolates were confirmed to be pathogenic to wheat. Furthermore, reisolation verified the identity of the isolates as causal organisms of spot blotch disease.

Although morphological studies are pivotal for the identification of plant pathogens at the genus level, molecular data are instrumental in the precise identification of fungal species [55]. The internal transcribed regions between ribosomal genes, such as 5.8S, have been studied widely to determine the molecular variability of the fungus [56]. In the present study, the primer pair ITS1/ITS4 amplified a fragment length of 600 bp. Verma et al. [23] also reported amplification at similar fragment lengths by the primer pair ITS1/ITS4 when working with isolates of *Bipolaris sorokiniana*. The use of ITS primers is preferred because the effectiveness of the primers is well documented [57]. Additionally, primers are increasingly being used for the study of fungal molecular ecology as well as evolutionary studies [58,59].

The phylogenetic analysis in the present study revealed a close evolutionary relationship between other isolates of *Bipolaris sorokiniana* and the isolates collected during the course of this study. Phylogenetic studies have been frequently used by other researchers to understand the biodiversity, evolution, ecology, and genomes of *B. sorokiniana* and similar species [59].

## 5 Conclusions

The results of this study provide significant insights into the aggressive nature of Bipolaris sorokiniana and the importance of spot blotch disease. It is a major disease of wheat, particularly in warm and humid regions of South Asia, including India, Bangladesh, Pakistan and Nepal. In this study, different isolates of *B. sorokiniana* were collected from different locations in West Bengal, India, to determine the pathogenic species of *B. sorokiniana* at these locations. The pathogenic species were identified on the basis of comprehensive morphological studies supplemented with ITS-rDNA sequence analysis and pathogenicity. Finally, validation was performed on the basis of the results of the molecular study. Among the 12 collected isolates from the studied location, the greatest radial growth was observed for BSC11 (50.07 mm), whereas the lowest growth was recorded for BSC9 (6.47 mm) at 96 h after inoculation. In pathogenicity assays, BSC11 presented the highest AUDPC (380.05) and PDI among the isolates at 20 dai (38.33 %). This study established BSC11 as a highly virulent pathogen isolate that can be used further for comprehensive analysis of *wheat‒Bipolaris sorokiniana* interactions. Our findings highlight the necessity for continuous monitoring and development of resistant cultivars to manage the impact of this pathogen effectively. Additionally, this study contributes to the broader understanding of host‒pathogen interactions and reinforces the need for the adoption of proactive management strategies through the deployment of varieties at certain time intervals to ensure food security and sustainable crop production. In the future, the molecular mechanisms underlying disease development can be studied, as can the development of integrated management strategies, which could help mitigate the risks posed by *Bipolaris sorokiniana*.

## CRediT authorship contribution statement

**Sunanda Chakraborty:** Writing – original draft, Visualization, Validation, Software, Methodology, Investigation, Formal analysis, Data curation, Conceptualization. **Sunita Mahapatra:** Writing – original draft, Visualization, Validation, Supervision, Methodology, Investigation, Conceptualization. **Anubhab Hooi:** Writing – original draft, Visualization, Validation, Methodology, Investigation, Conceptualization. **B. Teja Bhushan:** Writing – original draft, Visualization, Validation, Methodology, Investigation, Conceptualization. **Mansour I. Almansour:** Writing – review & editing, Software, Project administration, Funding acquisition, Formal analysis, Data curation. **Mohammad Javed Ansari:** Writing – review & editing, Software, Project administration, Funding acquisition, Formal analysis, Data curation. **Akbar Hossain:** Writing – review & editing, Writing – original draft, Supervision, Resources, Funding acquisition, Formal analysis, Data curation.

## Informed consent statement

Not applicable.

## Institutional review board statement

Not applicable.

## Data availability

The data will be available upon reasonable request to the corresponding authors.

## Funding

The study was funded by the Department of Plant Pathology, Bidhan Chandra Krishi Viswavidyalaya, Mohanpur, Nadia, WB, India. The project was also supported by Researchers Supporting Project number (RSPD2024R900), 10.13039/501100002383King Saud University, Riyadh, Saudi Arabia.

## Declaration of competing interest

The authors declare that they have no known competing financial interests or personal relationships that could have appeared to influence the work reported in this paper.
